# The Case for Telemedicine-Enhanced Nighttime Staffing in a Neuro-ICU

**DOI:** 10.1097/CCE.0000000000001231

**Published:** 2025-03-05

**Authors:** Belinda L. Udeh, Nicolas R. Thompson, Ryan D. Honomichl, Brittany R. Lapin, Irene L. Katzan, Lori Griffiths, Joao A. Gomes

**Affiliations:** 1 Neurological Institute Center for Outcomes Research and Evaluation, Cleveland Clinic, Cleveland, OH.; 2 Center for Population Health Research, Cleveland Clinic, Cleveland, OH.; 3 Department of Quantitative Health Sciences, Cleveland Clinic, Cleveland, OH.; 4 Neurological Institute, Cleveland Clinic, Cleveland, OH.

**Keywords:** intensive care units, neurology, outcome assessment—healthcare, telemedicine, workforce

## Abstract

**IMPORTANCE:**

This study compares the health outcomes and healthcare utilization of two staffing models for specialized neuro-ICU (NICU): a 24/7 intensive staffing (IS) model and a daytime 12-hour intensivist model with 12-hour nocturnal telemedicine-enhanced (TE) coverage. The IS model was studied from July 2016 to June 2017. The TE model was studied during the implementation period from July 2017 to June 2018.

**OBJECTIVES:**

To compare the health outcomes and healthcare utilization of two staffing models for a specialized NICU.

**DESIGN:**

Retrospective cohort study.

**SETTING AND PARTICIPANTS:**

NICU with 24 beds in a 1200-bed urban, quaternary care, academic hospital in Northeast Ohio. Participants were critically ill patients with primary neurologic injuries admitted to the NICU between July 2016 and June 2018.

**MAIN OUTCOMES AND MEASURES:**

Multivariable logistic, and negative binomial regression analysis compared the following outcomes: mortality, ICU length of stay (LOS), hospital LOS, and ventilator days. Demographics and patient characteristics, including Acute Physiology and Chronic Health Evaluation scores, were used in model adjustments.

**RESULTS:**

Three thousand seventy-three patients were studied: *n* equals to 1542 IS (average age 61 yr [sd 17], 49% female, 73% White race) and *n* equals to 1531 TE (average age 62 yr (sd 17), 49% female, 70% White race). The TE model required less staff than IS model (5 vs. 9 staff intensivists), respectively. Compared with IS, the TE cohort had similar demographics and clinical indications, although the groups differed on the distribution of the body systems necessitating ICU admission. TE model was protective of ICU mortality compared with IS model (odds ratio = 0.59; 95% CI, 0.43–0.82; *p* = 0.002). However, TE was associated with a 10% increase in ICU LOS (incident rate ratio [IRR] = 1.10; 95% CI, 1.03–1.18; *p* = 0.006) and a 13% increase in total LOS (IRR = 1.13; 95% CI, 1.06–1.20; *p* < 0.001). There was no difference in ventilator days between groups.

**CONCLUSIONS AND RELEVANCE:**

The availability of critical care staff is not keeping pace with demand, especially in specialized ICUs, including NICU. The TE model required fewer staff with similar clinical outcomes. This is a preliminary study highlighting that alternate specialized ICU staffing models could require fewer labor requirements while still maintaining quality of care. Further research is required to assess the true impact of LOS differences and examine the impact of these models on physician burnout and retention. This new understanding would provide additional guidance on ICU staffing options and telemedicine costs to hospitals, ensuring efficient and effective resource allocation as ICU demands continue to increase.

**KEY POINTS**

**Question:** Can a telemedicine-enhanced (TE) model for nocturnal coverage in a specialized ICU provide the same quality of care as an intensive staffing (IS) model?

**Findings:** This retrospective cohort study of 3073 patients found that the TE model was protective for ICU mortality as compared with IS, but was associated with a longer ICU and overall Length of stay.

**Meaning:** A TE model can provide at least equivalent quality of care to an IS model. Further research will help understand the economic implications and provide hospitals with future ICU staffing guidelines.

With an aging population and continued medical advancements, society’s demand for intensive care (IC) increases at alarming rates (1–4). Despite increasing age, illness severity, and care level required for critically ill patients, complications and mortality rates have declined (5). Improvements inpatient outcomes are attributed to dedicate ICUs in hospitals staffed by highly trained physicians, nurses, and other specialized health professionals (6), with higher-intensity staffing models showing more favorable outcomes (7). However, despite research showing that adopting the Leapfrog group ICU physician staffing standard is associated with decreased length of stay (LOS) and significant reductions in ICU mortality, less than one-half of all U.S. hospitals can provide this staffing level (8, 9).

Two main factors constrain our ability to meet future societal demands for IC: cost and healthcare providers. From 2000 to 2010, the cost of ICU beds per day increased by 61% ($2669–4300), and the total U.S. IC costs increased by 92% ($56.6 billion–108 billion) (10). In 2010, this represented 13.2% of hospital costs, 4.1% of all U.S. healthcare costs, and 0.72% of our gross domestic product (10). In addition, the current and projected future workforce is not sufficient to meet the demand (1, 11). By 2030, only 65% of the required intensivist hours will be available (1). Starting in December 2019, the COVID-19 pandemic exacerbated us to address these shortfalls. This virus posed unprecedented demand for healthcare services, none more so than IC (12, 13). The pandemic highlighted the critical role of IC services in a health system and the fact that meeting the demand is untenable at current staffing levels. To maintain patient outcomes, how ICU care is delivered must be reimagined.

In specialty ICUs, challenges are magnified. For instance, neurointensivists represent only a small fraction of critical care physicians in the United States. Still, their presence is associated with improved clinical outcomes in patients with devastating brain and spinal injuries (14, 15). In addition, many neurologic emergencies require the immediate availability of these experts. The adage “time is brain” highlights this need. Improving medical care increases the demand and complexity of neuro-ICU (NICU) services, and the population in need expands with new treatments (16).

Debate is continuing regarding the level and access to intensivists. Although 24/7 coverage may benefit subsets of ICU patients, limited numbers and increased intensivist costs may make 24/7 models untenable. Accordingly, different models of nocturnal ICU coverage have been proposed, including the use of telemedicine (17, 18).

Two common telemedicine models include a mobile telemedicine unit on a cart supplementing nocturnal care from a home-based on-call physician; or a nocturnal virtual ICU with fully connected bed monitoring, protocol guided care staffed by physicians and nurses at an off-site-hub. This second model has demonstrated significant improvement in clinical outcomes, including reduced nighttime mortality (19), but the required resource investment is substantial. This model commonly results in hospitals contracting with third parties to provide virtual ICU clinical services resulting in ongoing licensing costs. A mobile telemedicine unit on a cart requires less costs without the need for third-party contractors. However, it is provided the continuation of care by neurointensivists in the nighttime hours is vital in a NICU.

Our health system transitioned from a nocturnal intensivist model to a telemedicine-enhanced (TE) model, allowing for the comparison of clinical outcomes in a large, specialized NICU. Our study aimed to compare clinical outcomes and healthcare resource utilization between a nocturnal intensivist model and a TE model in a NICU.

## MATERIALS AND METHODS

This retrospective study compares the health outcomes and healthcare utilization of two staffing models for specialized NICU: A 24/7 intensive staffing (IS) model and a daytime 12-hour intensivist model with 12-hour nocturnal TE coverage. All procedures followed were in accordance with the ethical standards of our organization’s institutional review board (Number 21-464, “Telemedicine-enhanced Nighttime Staffing in a Neurointensive Care Unit,” approved April 15, 2021). Because the study consisted of analyses of preexisting data, the requirement for patient informed consent was waived. The study procedures followed were in accordance with the ethical standards of the responsible committee on human experimentation and with the Helsinki Declaration of 1975.

### Setting and Sample

We conducted this study in the 24-bed NICU of a 1200-bed urban, quaternary care academic hospital in Northeast Ohio. The study consisted of two time periods in which each staffing model was implemented. IS, evaluated July 2016 to June 2017, included nine neurointensivists, eight advanced practice nurses (APNs), trainees, and other critical care staff provided 24/7 in-house coverage. TE, evaluated from July 2017 to June 2018, included 5 neurointensivists and 10 APNs (i.e., a reduction of four staff physicians and the addition of two APPs). This model provided 12-hour daytime in-house coverage by a neurointensivist and 12 hours of nighttime coverage by an APN and trainees. The critical care team could request telemedicine consultations at their discretion, with video support provided by an on-call neurointensivist working remotely. Pre-established criteria for activating the telemedicine platform were defined to address situations in which virtual consultation would enhance patient care. These included acute changes in neurologic status, interpretation of bedside EEG monitoring for potential subclinical seizures, assessment of abnormal cardiac rhythms, and facilitation of family discussions. Although staff and team retained flexibility to initiate telemedicine beyond these specified criteria, traditional phone consultations between staff physicians, fellows, and advanced practice providers remained the primary mode of communication.

All other staffing remained the same between models. All patients admitted to the NICU during the two time periods were included. Only data from the first admission were retained for patients with multiple admissions. Approximately 30% of ICU patients are classified as low-risk monitors. They require monitoring, including neuro checks, but do not require additional ICU interventions, such as mechanical ventilation or vasoactive drugs. Although not considered to be actively treated, we included these patients as they are still managed by the ICU team.

### Outcomes and Covariates

Data from the Acute Physiology and Chronic Health Evaluation (APACHE) clinical information system was used. Primary outcomes were ICU mortality, ICU LOS, overall hospital LOS, and number of ventilator days. Because of the limitation of APACHE data, we only recorded ventilator days in cases where ventilation was initiated on day 1. The APACHE tools are prognostic scoring systems used for patients in intensive care and are based on available data from the first 24 hours of ICU admission. In our study, the APACHE III score was used as a measure of disease severity based on age, acute physiology score, and chronic health conditions (20). APACHE IV scores are calculated as risk predictions for ICU mortality, ICU LOS, and hospital LOS and include age, acute physiology score, emergency surgery, ICU readmission, mechanical ventilation, Glasgow Coma Scale score, seven chronic health conditions, admission source, and admission diagnosis (21, 22). Our model covariates included demographics, hospital characteristics, and APACHE scores (based only on day 1 of the admission).

### Statistical Methods

We used descriptive statistics to compare the cohorts’ demographics and clinical characteristics. Frequency count with percentage was used to present categorical data and mean with sd or median with interquartile range was used to present continuous data. As appropriate, we used chi-square tests to compare categorical variables and *t* tests (parametric) or Mann-Whitney *U* tests (nonparametric) to compare continuous variables across cohorts.

Using multivariable regression models, we assessed the effect of staffing models (IS vs. TE) on ICU LOS, overall hospital LOS, and the number of ventilator days. Logistic regression analysis modeled the risk of ICU mortality. For the mortality and LOS models, the related national predicted mortality probability, or LOS, based on the APACHE IV score, was used for either ICU or hospital to control individual differences in risk severity. For the model predicting ventilator days, the APACHE III score was used as a covariate instead, as the need for ventilatory support is included in the calculation of APACHE IV scores. As the APACHE scores include demographics and comorbidities, a second model was constructed that adjusted for the following variables: age, sex, race, diabetes, chronic obstructive pulmonary disease (COPD), immune suppression, (leukemia, myeloma, non-Hodgkin’s lymphoma, solid tumor with metastasis), chronic dialysis, cirrhosis, hepatic failure, AIDS, emergency surgery, admission diagnosis (neurologic vs. other), sepsis, low-risk monitor. For all non-mortality outcomes, we followed the practice of Thomas et al (23) and looked only at surviving patients to avoid truncation of outcome data due to mortality. ICU and hospital LOS were modeled using negative binomial regression. We modeled the number of days the patient was on a ventilator using zero-inflated negative binomial regression. This model has two parts: logistic regression examining the probability of being ventilated vs. not, and negative binomial regression examining the number of ventilator days in ventilated patients. Odds ratios (ORs) and incidence rate ratios (IRRs) were produced for the former and latter models, respectively.

For primary outcomes analysis, we used R, Version 4.3.1 (R Foundation for Statistical Computing, Vienna, Austria) (24), MASS package (25) for negative binomial models, and the pscl package (26) for zero-inflated negative binomial models. We identified and summarized other outcomes relevant to healthcare utilization. Statistical significance was established at *p* value of less than 0.05.

## RESULTS

A total of 3073 adult patients were included in our study: 1542 IS model (50.2%) and 1531 TE cohort (49.8%). After the transition to the TE cohort, four fewer physicians were staffed in the NICU but two additional nurse practitioners were added, as compared with the IS time period. During the study period for TE cohort, the median number of activations of the mobile telehealth monitoring unit was 10.5 per month.

Patients of the TE cohort were slightly older than the patients in IS model (62.4 ± 17.1 vs. 60.9 ± 16.9 yr, respectively, *p* = 0.012), and the two groups differed in the proportion with COPD (17% IS vs. 20% TE, *p* = 0.033), and distribution of affected body systems necessitating ICU admission (*p* = 0.006) (**Table 1**). Otherwise, the cohorts were comparable.

**TABLE 1.** Comparison of Descriptive Statistics Between Staffing Model Cohorts, *n* = 3073

Variable	Nighttime Intensive, *N* = 1542	Telemedicine, *N* = 1531	*p*
Age (yr), mean (sd)	60.9 (16.9)	62.4 (17.1)	0.012
Female, *n* (%)	758 (49.2)	748 (48.9)	0.896
Race, *n* (%)			0.203
White	1104 (73.1)	1054 (70.2)	
African American	366 (24.2)	400 (26.6)	
Other	41 (2.7)	48 (3.2)	
Diabetes, *n* (%)	348 (22.6%)	356 (23.3%)	0.683
Chronic obstructive pulmonary disease, *n* (%)	264 (17.1%)	309 (20.2%)	0.033
Immune suppression, *n* (%)	154 (10.0%)	170 (11.1%)	0.343
Leukemia, myeloma, non-Hodgkin’s lymphoma, solid tumor with metastasis, *n* (%)	154 (10.0%)	172 (11.2%)	0.287
Chronic dialysis, *n* (%)	46 (3.0%)	50 (3.3%)	0.729
Cirrhosis, *n* (%)	44 (2.9%)	59 (3.9%)	0.150
Hepatic failure, *n* (%)	24 (1.6%)	32 (2.1%)	0.332
AIDs, *n* (%)	7 (0.5%)	6 (0.4%)	0.998
Emergency surgery, *n* (%)	62 (4.0)	54 (3.5)	0.533
Admission diagnosis by organ system, *n* (%)			0.005
Neurologic	1070 (69.4)	1163 (76.0)	
Cardiovascular	237 (15.4)	178 (11.6)	
Respiratory	93 (6.0)	70 (4.6)	
Trauma	72 (4.7)	70 (4.6)	
Gastrointestinal	28 (1.8)	15 (1.0)	
Hematologic	17 (1.1)	18 (1.2)	
Metabolic/endocrine	14 (0.9)	8 (0.5)	
Genitourinary	9 (0.6)	7 (0.5)	
Musculoskeletal/skin	2 (0.1)	2 (0.1)	
Sepsis, *n* (%)	44 (2.9%)	54 (3.5%)	0.337
Low-risk monitor patient, *n* (%)	601 (39.0%)	648 (42.3%)	0.064
APACHE III Score, median (q1, q3)	47 (34, 64)	48 (33, 64)	0.694
APACHE IV hospital mortality prediction, median (q1, q3)	0.04 (0.02, 0.13)	0.05 (0.02, 0.14)	0.167
APACHE IV ICU mortality prediction, median (q1, q3)	0.02 (0.01, 0.07)	0.03 (0.01, 0.07)	0.314
APACHE IV hospital LOS prediction, median (q1, q3)	7.9 (5.9, 10.5)	7.7 (5.9, 10.4)	0.636
APACHE IV ICU LOS prediction, median (q1, q3)	3.2 (2.3, 5.1)	3.1 (2.3, 4.9)	0.347
ICU mortality, *n* (%)	150 (9.7)	96 (6.3)	< 0.001
ICU LOS (d), median (q1, q3)	2.1 (1.1, 4.3)	2.2 (1.2, 4.5)	0.022
Hospital LOS (d), median (q1, q3)	7.0 (3.3, 13.3)	7.5 (3.7, 14.6)	0.011
Ventilator Use, *n* (%)	525 (34.0%)	473 (30.9%)	0.068
Ventilator days, median (q1, q3)	2 (1, 5)	3 (1, 6)	< 0.001

APACHE = Acute Physiology and Chronic Health Evaluation, LOS = length of stay, q = quartile.

There were 150 ICU deaths (9.7%) for IS, compared with 96 ICU deaths (6.3%) for TE (*p* < 0.001) (Table 1). The distributions of ICU death by affected body system and staffing model cohort were not significantly different (*p* = 0.739) (**Table 2**). After controlling for APACHE IV predicted mortality risk score, being in the TE cohort was protective for ICU mortality compared with IS (OR = 0.59; 95% CI, 0.43–0.82; *p* = 0.002) (**Table 3**). Similarly, after controlling for patient and clinical characteristics, ICU mortality was lower in the TE cohort (OR = 0.59; 95% CI, 0.45–0.78; *p* < 0.001).

**TABLE 2.** Frequency and Percentage of ICU Mortality by Staffing Model Cohort and Admission Diagnosis by Organ System

Admission Diagnosis by Organ System	Nighttime Intensive	Telemedicine
Neurologic	94/1070 (8.8%)	61/1163 (5.2%)
Cardiovascular	37/237 (15.6%)	27/178 (15.2%)
Respiratory	7/93 (7.5%)	6/70 (8.6%)
Trauma	6/72 (8.3%)	2/70 (2.9%)
Gastrointestinal	3/28 (10.7%)	0/15 (0.0%)
Hematologic	1/17 (5.9%)	0/18 (0.0%)
Metabolic/endocrine	1/14 (7.1%)	0/8 (0.0%)
Genitourinary	1/9 (11.1%)	0/7 (0.0%)
Musculoskeletal/skin	0/2 (0.0%)	0/2 (0.0%)

The distribution of ICU mortality by staffing model cohort and organ system was not significantly different (*p* = 0.739).

**TABLE 3.** Estimates for Telemedicine Staffing Model (Versus Nighttime Intensive Staffing Model) in Two Adjusted Models

Outcome	Model 1 (Acute Physiology and Chronic Health Evaluation Only)		Model 2 (Patient/Clinical Covariates)	
	OR (95% CI)	*p*	OR (95% CI)	*p*
ICU mortality	0.59 (0.43–0.82)	0.002	0.59 (0.45–0.78)	< 0.001
Ventilator use (joint model)	0.78 (0.59–1.02)	0.073	1.09 (0.33–3.57)	0.884
	IRR (95% CI)	*p*	IRR (95% CI)	*p*
ICU LOS	1.10 (1.03–1.18)	0.006	1.08 (1.00–1.17)	0.037
Hospital LOS	1.13 (1.06–1.20)	< 0.001	1.09 (1.02–1.16)	0.009
Ventilator days (joint model)	1.11 (0.92–1.33)	0.270	1.01 (0.84–1.21)	0.943

IRR = incident rate ratio, LOS = length of stay, OR = odds ratio.

ICU mortality was modeled in a logistic regression model with estimates for telemedicine staffing presented as OR with 95% CI. ICU LOS and hospital LOS were modeled in negative binomial models with estimates presented as an IRR with 95% CI. Ventilator use and days were jointly modeled using zero-inflated negative binomial regression models including a logistic regression model for ventilator use and a negative binomial regression model for days for those who were ventilated. Model 1 adjusted for Acute Physiology and Chronic Health Evaluation (APACHE) score: ICU mortality model used APACHE IV ICU mortality prediction, ICU LOS model used APACHE IV ICU LOS prediction, hospital LOS model used APACHE IV hospital LOS prediction, Ventilator days model used APACHE III. Model 2 adjusted for age, sex, race, diabetes, Chronic obstructive pulmonary disease, immune suppression (leukemia, myeloma, non-Hodgkin’s lymphoma, solid tumor with metastasis), chronic dialysis, cirrhosis, hepatic failure, AIDS, emergency surgery, admission diagnosis (neurologic vs. other), sepsis, low-risk monitor.

The median ICU LOS for IS was 2.1 days (q1, q3: 1.1–4.3), compared with 2.2 days (q1, q3: 1.2–4.5) for TE, *p* = 0.022. After controlling for APACHE IV predicted LOS, via negative binomial regression, TE was associated with a 10% increase in total days in the ICU, compared with IS (IRR = 1.10; 95% CI, 1.03–1.18; *p* = 0.006) (Table 3). After adjusting for patient and clinical characteristics, TE was associated with an 8% increase in total ICU days (IRR = 1.08; 95% CI, 1.00–1.17; *p* = 0.037).

For overall hospital LOS, IS had a median LOS of 7.0 days (q1, q3: 3.3–13.3), and TE had a median of 7.5 days (q1, q3: 3.7–14.6), *p* = 0.011. After adjustment for APACHE IV hospital LOS, TE was associated with a 13% increase in LOS compared with IS (IRR = 1.13; 95% CI, 1.06–1.20; *p* < 0.001) (Table 3). Results were similar after adjusting for patient and clinical characteristics (IRR = 1.09; 95% CI, 1.02–1.16; *p* = 0.009).

For IS, there were 525 (34.0%) patients ventilated on their first day in the ICU, in contrast to 473 (30.9%) for TE (*p* = 0.068) (Table 1). After controlling for Apache III scores, and patient and clinical covariates, there was not a significant difference between TE and IS regarding odds of being ventilated (Table 3). For those who were ventilated, the median ventilator days were significantly higher in the TE cohort (3 [q1, q3: 1–6]) compared with the IS group (median 2 [q1, q3: 1–5]), *p* value of less than 0.001. After adjustment, there was not a significant difference between TE and IS cohorts (model 1 IRR = 1.11; 95% CI, 0.92–1.33; *p* = 0.27).

## DISCUSSION

The main findings of our study are that the TE model in a specialty ICU appears to be associated with decreased ICU mortality but prolonged ICU LOS and hospital LOS compared with an IS staffing model. With increased utilization of telemedicine platforms because of technological advances and the need for more efficient use of ICU resources, studies continue reporting outcomes of different staffing strategies (18). However, nearly all these studies have focused on general ICUs, not specialized ones, including NICUs. To our knowledge, this is the most extensive study of a specialized ICU.

Although prior investigations assessing the effect of nighttime intensivist staffing models on mortality and LOS have shown no clear advantage over high-intensity daytime staffing (27–29), previous studies examining the association between tele-ICU arrangements and clinically relevant outcomes have reported mixed results (30, 31). The differences in study results are likely due to patient population characteristics, ICU setting, practice patterns for telemedicine activations, and simultaneous implementation of other interventions to optimize patient outcomes.

Our findings agree with that of Wallace et al (28) and Kerlin et al (27) in that the nighttime presence of a neurointensivist on site did not translate into improved mortality benefits (compared with the telemedicine model). ICU mortality was lower in our telemedicine cohort (6.3% vs. 9.7%). Although McCambridge et al (31), Breslow et al (30), Fusaro et al (32), and Udeh et al (33) reported improved mortality outcomes with the implementation of telemedicine-based intensivist programs, the settings, and circumstances were different from ours. Despite this association, it is difficult to conclude that a nighttime remote telemedicine program is associated with decreased mortality compared with the onsite presence of neurointensivists. Likely, other factors (i.e., ongoing quality and process improvement interventions) may have played a role. Indeed, a multicenter retrospective study by Kerlin et al (34) suggests that ICUs without physicians at night reduce hospital mortality because of differences in end-of-life care practices. At the very least, however, the switch from IS to TE coverage did not result in worse mortality outcomes.

Similarly, mixed results have been reported when assessing the association between ICU LOS or hospital LOS and ICU staffing models. In most of our models, our study demonstrated slightly longer ICU LOS or hospital LOS for the TE cohort compared with the IS cohort. Although high-intensity ICU staffing is associated with decreased LOS (7), the addition of nighttime intensivists failed to show any significant improvement in LOS in a recent meta-analysis (27). The use of teleintensivist coverage decreased LOS in at least one study (30) but not in others (31, 35). Although the potential financial implications of prolonged LOS cannot be ignored, it is difficult to ascribe this finding exclusively to the change in the staffing model. Other factors (i.e., floor/step-down bed availability, deferment of transfer decisions to daytime hours, and ease of discharge to rehab or long-term care facilities) may contribute to the increased LOS observed.

Furthermore, it has long been debated how to measure ICU LOS accurately and how much of an impact total LOS truly has on the cost of hospital admission (36, 37). Taheri et al (37) conducted a micro-accounting review of over 12,000 discharges at their academic medical center with an LOS longer than 4 days. Costs were subcategorized by category and day. For surgical patients, the incremental cost on the last full day of their hospital stay was just $420, 2.4% of their total costs. For non-surgical patients, it was $432, 3.4% of their total costs. For most hospitals, over 60% of costs are fixed, and patients incur most variable costs on their first three days of admission. In this study, the average total LOS for the 24/7 and TE models was approximately 10 and 11 days, respectively. Although shorter LOS improves hospital efficiency, reduces the potential for hospital strain, and reduces the likelihood of hospital-acquired complications, including infections and falls (38). Although our study did show a difference in LOS, this is only a preliminary study. Further studies, including a prospective trial, would be required to determine the true cost difference between these two ICU staffing models attributable to LOS differences (18). The increased hospital costs incurred with the TE model of care from the increased LOS are partially offset by the reduction in staffing costs. This model required four fewer physicians and two more nurse practitioners to staff the NICU compared with the 24/7 model. With an activation rate of 10.5 occasions per month, one mobile telehealth monitoring unit is sufficient for a specialized ICU of this size. Its portability, simplicity, and ability to operate within an ICU make it a relatively cheap investment, especially compared with a fully functioning eICU setup for an ICU where each bed requires significant infrastructure investment and the services provided by contract through a third party. As mentioned previously, the actual economic evaluation of the two staffing models would require more robust measures of the clinical and healthcare utilization outcomes relative to each strategy’s labor and capital costs.

Aside from the potential clinical and economic implications identified in this study, other potential benefits include improved staff well-being and reduced burnout. It is well understood that healthcare professionals, in general, are at an increased risk of burnout, magnified and exacerbated by the COVID-19 pandemic. Still, intensivists are estimated to have the highest burnout rates of any specialty (39). The consequences of burnout have been well documented, including quality and cost of care, patient safety, and clinician turnover, with the national consensus that it is an issue that must be addressed (40–42). A significant contributor to increased rates of burnout for intensivists is work schedules that include nighttime hours (43, 44). Potentially, the TE staffing model could require less nighttime intensivist scheduling and reduced intensivist demand in the long term. Although beyond the scope of this study, identifying and quantifying any differences in the rates of intensivist satisfaction and burnout would further help determine any value differences in the staffing models.

Although this was the most extensive known study for a specialized ICU, it was retrospective, with notable limitations unable to be addressed. First, there are potential confounders we were not able to address such as bed availability, deferment of transfer decisions to daytime hours, ease of discharge to rehab or long-term care facilities, differences in nursing personnel due to normal turnover rates, modifications to electronic health record documentation templates, and additional factors. We did include APACHE risk scores, which include many relevant patient characteristics. We additionally constructed adjusted models including patient and clinical characteristics including age, sex, race, comorbidities, and other important confounding variables, which provided evidence of the robustness of our findings. Other possible confounders include any changes in clinical protocols or services during these study periods, however, no other changes were made during this aside from the staffing models. Our study did not include a “phasing-in” or wash-out window when the staffing models changed. The number of times the telehealth service was activated per month was known, but we could not determine how many times the physician on-call was contacted by phone or required to come to the hospital to provide care. This information and physician satisfaction would help understand the actual value of the telehealth intervention, especially relative to burnout and physician retention. A further limitation was we could only measure ventilator days for patients placed on a ventilator on the first day of ICU stay. In addition, the true cost impact of the increased ICU and total hospital LOS in patients in the telemedicine model was difficult to determine. Further research is required to calculate these LOS differences’ clinical and economic implications. Finally, our telemedicine model, with approximately 10 activations monthly, employed specific practice patterns that may differ from other hospital systems. Consequently, these findings may not be generalizable to telemedicine programs with varying frequencies of use and activation protocols. The U.S. healthcare system is at a critical point in providing quality IC healthcare. The population is aging, technologies are increasing, the intensivist workforce is continuing to fall behind the demand, and we must always be prepared to handle worldwide health crises like COVID-19. For specialist ICUs, including NICUs, these issues are magnified. The staffing model for this study replaced half of its physicians with APNs and used a mobile telemedicine cart to support APNs during nighttime hours. This preliminary study highlights that alternate specialized ICU staffing models could require fewer labor requirements while still maintaining the quality of care. Prospective studies are needed to understand the true impact of the differences in healthcare resources, clinical outcomes, and physician benefits, and provide additional guidance to hospitals on ICU staffing options relative to staffing and telemedicine technology costs.
